# Evaluating the Impact of Psychiatric Disorders on Preoperative Pain Ratings, Narcotics Use, and the PROMIS-29 Quality Domains in Spine Surgery Candidates

**DOI:** 10.7759/cureus.12768

**Published:** 2021-01-18

**Authors:** Zachary Christian, Olusoji Afuwape, Zachary D Johnson, Emmanuel Adeyemo, Umaru Barrie, Luke J Dosselman, Mark N Pernik, Kristen Hall, Salah G Aoun, Carlos A Bagley

**Affiliations:** 1 Neurological Surgery, University of Texas Southwestern Medical Center, Dallas, USA; 2 Neurosurgery, University of Texas Southwestern Medical Center, Dallas, USA

**Keywords:** psychiatric disorder, spine surgery, pain interference, pain rating, opioid use, outcome measures, promis 29, preoperative assessment

## Abstract

Objective

We aimed to study the relationship between psychiatric Disorders (PD), preoperative pain, and opioid medication intake, as well as the quality of life patient-reported outcome measures using the Patient-Reported Outcomes Measurement Information System 29 (PROMIS-29) questionnaire, during the 30-day interval preceding surgery, in a consecutive series of patients who were scheduled to undergo surgical spine procedures. We hypothesized that PD could affect preoperative narcotic use and pain interference in a fashion that was not linearly associated with preoperative pain in spine surgery candidates.

Methods

The records of consecutive adult patients who underwent elective spinal surgery between October 2016 and August 2017 at a single institution were reviewed. We included patients who underwent preoperative pain assessment within 30 days prior to their planned surgery using the PROMIS-29 questionnaire. Patients with PD were compared to controls.

Results

A total of 117 patients matched our criteria. The average rating of pain intensity was notably higher in the PD group as compared to controls (p=0.004). The PD group had more patients complaining of high pain levels (>6) as compared to the control group (p=0.026). Controls with high pain levels had a greater incidence of preoperative narcotic use as compared to the low-pain cohort (p=0.029). However, there was no difference in the actual dose of daily narcotic medication taken between the PD and control groups (P=0.099) or between the low- and high pain score groups in the control (p=0.291) and PD (p=0.441) groups, respectively. Patients with PD and higher pain ratings seemed to have a higher incidence of anxiety (p=0.005) and depression (p<0.001). That was not the case for controls.

Conclusions

PDs may impact the degree of preoperative pain interference and the intake of narcotic medication independently from pain intensity ratings.

## Introduction

Spine surgery candidates have been shown to have higher exposure to opioid medication than the general population [1]. A depressive disorder is also an independent risk factor for opioid abuse [2-3], and a positive correlation between the preoperative ratings of depression and anxiety and the postoperative perception of pain has been established [4-7]. Psychiatric disorders (PD), such as affective disorder, anxiety disorder, and stress-related disorders in surgical patients, have also been associated with increased postoperative complications, length of stay, readmission rates, and poor long-term outcomes [4,8-10]. However, the relationship between PD and a systematic assessment of the preoperative quality of life using the Patient-Reported Outcomes Measurement Information System 29 (PROMIS-29) has not been previously explored in spine surgery candidates. Shedding light on this relationship may allow providers to better understand the failure of some patients to respond to nonoperative treatment, which often creates an indication for surgery. It may also assist surgeons to better optimize patients preoperatively by treating their stress, anxiety, and depression, thus maximizing the benefit brought by a surgical resolution and increasing patient satisfaction.

We aimed to study the relationship between PD, preoperative pain and opioid medication intake, and quality of life patient-reported outcome measures, during the 30-day interval preceding surgery, in a consecutive series of patients who underwent surgical spine procedures. We hypothesized that PD could influence preoperative narcotic intake, as well as ratings of pain interference with activities of daily living independently from pain scores in surgical spine candidates.

## Materials and methods

Protocol

The study protocol was approved by our institutional review board (STU# 102017-011). This was a retrospective review of prospectively collected data at a single-center institution that included consecutive patients who underwent elective spine surgery between October 2016 and August 2017. We only included patients who underwent a preoperative pain assessment within 30 days prior to their planned surgery, as we wanted to include the most recent assessment prior to surgical intervention. Patient data were prospectively collected in our Spine Clinical Outcome Registry (SCORe) and retrospectively reviewed using our electronic medical record system for this analysis. Patient consent was not required for retrospective data pooling, as patient data were deidentified once collected as is standard at our institution.

Population selection criteria

Spinal procedures included posterior cervical, anterior cervical, thoracic or lumbar short (≤4 segments) and thoracic or lumbar long (≥5 segments) decompression and/or fusion procedures. Inclusion criteria consisted of patients older than 18 years of age who underwent elective spine surgery and had available PROMIS-29 scores within 30 days prior to surgery. The PROMIS-29 questionnaire is a publicly available, validated instrument that has been shown to reliably assess patient well-being, mental and physical health, and overall quality of life [11-12].

The patients were divided into two groups. Patients who had received a psychiatric diagnosis of depression, anxiety, bipolar disorder, and/or post-traumatic stress disorder were labeled as part of the psychiatric disorder group. The remainder of the cohort served as the control group. All patients with psychiatric disorders were diagnosed, followed, and treated by a certified psychiatrist at our institution. All patients in our cohort received a psychiatric assessment prior to surgery.

Outcome measures

Perioperative Measures and Prognosis

Demographic and clinical variables were collected to ensure uniformity between the psychiatric disorder and control groups. Demographic variables included age, gender, and race. Perioperative outcome measures included preoperative narcotic, alcohol, and tobacco use and prior history of spine surgery. PROMIS-29 criteria included pain, depression, and anxiety ratings collected within 30 days prior to the spinal procedure using the pain intensity, pain interference, depression, and anxiety domains. Pain intensity was assessed on a 10-point numeric scale. High-intensity pain was defined as a score of >6, as it corresponds to “very severe” and “worst pain possible’ on the visual analog scale [13]. The pain interference, depression, and anxiety domains each consisted of four five-point Likert scales. We also collected postoperative data, including hospital length of stay, intra and postoperative complications, emergency department (ED) visits six weeks after surgery, and hospital readmissions within 30 days after surgery. Narcotic users were defined as patients who were prescribed any narcotic medication within 30 days prior to the spinal procedure. Preoperative narcotic medication dose was converted to the daily morphine equivalent dose to allow for statistical comparison in accordance with previously published validated data [13-14]. Daily narcotic doses were reported by the patients during their 30-day preoperative interview. We limited our analysis to patients seen at our office within 30 days prior to their planned surgery, as we believed that that the interview would reflect the mindset of a preoperative patient better than if it was performed at a greater interval from the date of surgery.

Statistical analysis

Descriptive statistics included mean and standard deviation (SD). For continuous variables, significance was assessed using a one-tailed t-test. Fisher’s exact test was used to compare dichotomous variables between groups and assess differences in incidence. Statistical significance was set at ɑ = 0.05. The software used for the statistical analysis was the Statistical Package for the Social Sciences (SPSS) version 25 (IBM Corp, Armonk, NY).

## Results

Patient demographics

A total of 117 patients were identified to be included in the study. The PD group consisted of 61 patients and the control group of 56 patients (Table 1). Most patients in the psychiatric disorder group were female (60.66%) while the control group consisted mostly of males (66.07%) (p=0.002). The majority of patients in both groups were White.

**Table 1:** Demographic characteristics and PROMIS-29 pain intensity responses for the study population PROMIS-29: Patient-Reported Outcomes Measurement Information System 29; ACDF: anterior cervical discectomy and fusion; PCDF: posterior cervical decompression and fusion; EtOH: ethanol

Value		Control Group, n=56	PD Group, n=61	p-value
Demographic Data, n (%)				
Age (SD)		59.91 (15.46)	61.48 (12.58)	
Gender	Male	n=37 (66.07%)	n=24 (39.34%)	0.002
	Female	n=19 (33.93%)	n=37 (60.66%)	0.002
Race	White	40 (71.43)	48 (78.69)	0.261
	Black	8 (14.29)	5 (8.20)	0.190
	Hispanic	4 (7.14)	5 (8.20)	0.366
	Other*	4 (7.14)	3 (4.92)	0.350
Smoking History		17 (30.3%)	22 (36.06%)	0.51
Surgery Type				
Lumbar Short		39 (69.64%)	40 (65.57%)	0.64
Lumbar Long		1 (1.79%)	0 (%)	0.44
ACDF		9 (16.07%)	15 (24.6%)	0.25
PCDF		6 (10.71%)	5 (8.2%)	0.64
Misc		1 (1.79%)	1 (1.64%)	0.95
EtOH History		26 (46.43%)	32 (52.46%)	0.52
History of Prior Spine Surgery		17 (30.36%)	25 (41%)	0.23
Pain Intensity		5.91	7.05	0.004

Clinical characteristics of the PD group

In the PD group, 49.18% of patients were diagnosed with depression, 36.07% with anxiety disorder, 1.64% with bipolar disorder, and 1.64% with post-traumatic stress disorder. There were 24 patients in the PD group (39.34%) with an unspecified mood disorder. Seventy-five percent of patients were on antidepressants, 37.5% on anxiolytics, and 4.17% on a mood stabilizer. All patients were included in our analysis.

Pain rating in the PD and control groups

The average rating of pain intensity was notably higher in the psychiatric disorder group as compared to the control group (7.05 vs 5.91, p=0.004) (Table 1). The PD group had significantly more patients complaining of high pain levels (>6) as compared to the control group (p=0.026) (Table 2).

**Table 2:** Baseline preoperative variables and patient outcomes by preoperative pain score category: Low-Pain (≤6 pain score) and High-Pain (>6 pain score) cohorts PROMIS-29: Patient-Reported Outcomes Measurement Information System 29; ACDF: anterior cervical discectomy and fusion; PCDF: posterior cervical decompression and fusion; EtOH: ethanol; ED: emergency department

	Control Group (n=56)			Psychiatric Disorder Group (n=61)		
	Low Pain Cohort (<=6 Pain Score)	High-Pain Cohort (>6 Pain score)	p-value	Low-Pain Cohort (≤6 Pain score)	High-Pain Cohort (>6 Pain score)	p-value
	33 (58.93%)	23 (41.07%)		19 (31.15%)	42 (68.85%)	0.026
Demographic Characteristics						
Male	21 (63.64%)	16 (69.57%)	0.325	8 (42.11%)	16 (62.00%)	0.336
Female	12 (36.36%)	7 (30.43%)	0.325	11 (57.89%)	26 (38.00%)	0.336
White	24 (72.72%)	16 (69.57%)	0.155	16 (84.21%)	32 (76.19%)	0.138
Black	1 (3.03%)	7 (30.43%)	0.006	0 (0%)	5 (11.90%)	0.012
Hispanic	4 (12.12%)	0	0.022	3 (15.79%)	2 (4.76%)	0.122
Misc	4 (12.12%)	0	0.022	0	3 (7.14%)	0.042
Age	56.67	64.57	0.029	58.37	62.88	0.112
PROMIS-29 Scores						
Pain Interference	11.73	15.52	0.004	10.73	17.94	<0.001
Anxiety	7	8.19	0.156	6.67	9.70	0.005
Depression	5.59	7.38	0.061	5.67	9.18	<0.001
Social History						
Overall Incidence of Narcotic Use	28 (50%)			31 (51%)		0.91
Incidence of Narcotic Use	13 (39.39%)	15 (65.22)	0.029	7 (36.84%)	24 (57.14)	0.086
Overall Average Daily MED	27.55			33.64		0.099
Average Daily MED	29.27	26.44	0.2920	32.43	33.99	0.441
Smoker	9 (27.27%)	8 (34.78%)	0.281	5 (26.32%)	17 (40.48%)	0.212
EtOH	16 (48.48%)	10 (43.48%)	0.359	11 (57.89%)	21 (50%)	0.311
Psychiatric Medication	NA	NA		17 (89.47%)	37 (88.10%)	0.449
History of Spine Surgery	9 (27.27%)	8 (34.78%)	0.281	18 (36.84%)	7 (42.86%)	0.354
Surgery Type						
Lumbar Short	19 (57.58%)	20 (86.96%)	0.006	9 (47.37%)	31 (73.81%)	0.023
Lumbar Long	0	1 (4.35%)	0.164	0	0	
ACDF	8 (24.24%)	1 (4.35%)	0.014	8 (42.11%)	7 (16.67%)	0.030
PCDF	5 (15.15%)	1 (4.35%)	0.083	2 (10.53%)	3 (7.14%)	0.343
Misc	1 (3.03%)	0	0.162	0	1 (2.38%)	0.162
Intraoperative outcomes						
Complications	0	0		0	0	
Average LOS	1.35	2.39	0.143	1.86	1.89	0.285
Postoperative outcomes						
30-day Readmissions	0	0		0	1 (2.38%)	0.162
Complications	2 (6.06%)	0	0.080	2 (10.53%)	2 (4.76%)	0.285
6-week ED visits	1 (3.03%)	1 (4.35%)	0.402	2 (10.53%)	2 (4.76%)	0.285

Preoperative characteristics and postoperative outcomes in the PD and control groups in the function of pain scores

Table 2 reports the mean difference in perioperative outcomes between control and psychiatric disorder groups when the two groups are further subdivided by preoperative pain intensity score into the “Low-Pain” and “High-Pain” cohorts.

Patient demographics

For both the PD and control groups, most Black patients were in the high-pain cohort (PD: 11.90% vs 0%, p=0.012; control: 30.43% vs 3.03%, p=0.006). Amongst controls, Hispanics fell into the low-pain cohort (2.18% vs 0%, p=0.022). White patients were similarly distributed between pain groups. The average age of controls in the high-pain cohort was greater than the low-pain cohort (64.57 vs 56.67, p=0.029), but that was not the case for PD patients.

Patient social and past medical variables

There was no difference in smoking history, ethanol use, and surgical history between patients with psychiatric disorders and controls (Table 1). This remained true when examining the low-pain and high-pain cohorts within these groups (Table 2). Controls with high pain had a greater incidence of preoperative narcotic use as compared to the low-pain cohort (65.22% versus 39.39%, p=0.029). However, there was no difference in the actual dose of daily narcotic medication taken in MED between the control and the PD groups (P=0.099) or between the low-pain score and high-pain score groups in the control (p=0.292) and PD (p=0.441) groups, respectively. In the PD cohort, there was no difference in psychiatric medication use prior to surgery between low pain and high pain groups (Table 2).

PROMIS-29 ratings

Patients with higher pain ratings appeared to have higher pain interference scores in both the control (p=0.004) and psychiatric disorder (p<0.001) cohorts. Patients with PD who had higher pain ratings seemed to have a higher incidence of anxiety (p=0.005) and depression (p<0.001). That was not the case for controls.

Procedural characteristics

In both the PD and control groups, patients who were scheduled to undergo lumbar short surgeries tended to report higher levels of preoperative pain (PD: 73.81% vs 47.37%, p=0.023; control: 86.96% vs 57.58%, p=0.006). Conversely, the majority of patients who had anterior cervical surgery planned reported lower levels of preoperative pain (PD: 16.67% vs 42.11%, p=0.030; control: 4.35% vs 24.24%, p=0.014).

Perioperative outcomes

The average length of postoperative hospital stay and the incidence of intraoperative and postoperative complications, emergency department visits within six weeks after surgery, and readmission rates 30 days postoperatively were not significantly different between the low-pain and high-pain cohorts (Table 2).

## Discussion

The objective of this study was to investigate the impact of PD on preoperative pain ratings, pain interference using the PROMIS-29 questionnaire, and preoperative narcotic use. The population we examined consisted of patients with symptomatic spine disease that underwent surgery at a single institution within 30 days after their preoperative visit and interview. Psychiatric disorders have been shown to negatively impact postoperative pain and outcomes after spine surgery [3,6,15], and preoperative anxiety has been associated with postoperative pain [5,7,16-18]. In addition, preoperative narcotic use has been identified as an independent predictor of narcotic consumption after surgery [19-20], and preoperative pain levels have been shown to influence pain after surgery [21-22]. However, the relationship between PD, preoperative pain, and narcotic use has not been fully explored, and those three factors may have a compounding impact on the postoperative perception of pain and on patient outcomes.

The PD and control cohorts had comparable demographic characteristics when examining race, smoking and alcohol consumption history, and the incidence of prior spine surgery. However, there was a preponderance of women in the PD group, which is consistent with previously published data [23-24]. PD patients reported higher levels of preoperative pain intensity as compared to controls (7.05 vs 5.91, p=0.004). This finding is interesting and could reflect the impact of a psychiatric disease on a patient’s perception of pain, specifically with spinal pathology. When further subdividing the PD and control cohorts into the “low-pain” and “high-pain” groups, ethnicity appeared to play a role in the perception of pain intensity. Black patients were more likely to report higher levels of pain intensity in both the PD and control groups, which agrees with reports by Campbell and Edwards who noted that amongst individuals with chronic pain, Black patients were more likely to report higher average pain intensity than other ethnic groups [25]. Hispanic patients in the control cohort all belonged to the “low-pain” category.

When examining the PROMIS-29 questionnaire domains, patients with higher pain scores appeared to have a higher incidence of pain interference in both the control and PD groups. However, it was only in the PD group that higher pain ratings were also associated with a higher incidence of depression and anxiety. It is important to note that the low and high-pain categories in both the PD and control groups were comparable in the incidence of risk factors that have been previously associated with depression and the worsened perception of pain such as smoking [26] and ethanol use [27]. Patients with PD in the low-pain and high-pain groups were also comparably medicated, and all subcategories had similar rates of prior spine surgery. These findings reinforce the concept of a close relationship between pain, anxiety, and depression, not only in the postoperative setting but also before surgery.

Previous publications have shown an increase in preoperative opioid use among spine surgery candidates, especially in patients with depression [1-2]. Our data add to this body of knowledge by clarifying that on average, patients with PD have a higher preoperative rating of pain, as well as a higher level of interference with activities of daily living. However, increased pain levels do not independently account for increased narcotic use given a similar overall prevalence between the control and the PD groups (Table 2). Interestingly, the incidence of preoperative narcotic use in controls seems to increase with higher levels of pain, but that is not the case in the psychiatric disorder group, which may reflect an intrinsic effect of the psychiatric disorder [28]. PD patients may also have a history of opioid use to control pain, making opioid cessation for lower levels of pain challenging. Goesling et al. proposed that depressed patients may be more likely to struggle with therapeutic opioid dependence or opioid-induced hyperalgesia, making it difficult to discontinue opioids even after improvement in joint pain [29]. Sullivan also noted that depressed patients seem to continue opioid use at lower pain intensity levels and higher levels of physical function than nondepressed patients [30]. This data is unsettling in the context of the opioid crisis plaguing the United States and leading to over 33,000 deaths every year.

Despite variable patterns of preoperative pain intensity, pain interference, and opioid consumption, the incidence of intraoperative complications, average length of hospital stay, postoperative complications, emergency department visits within six weeks, and readmission 30 days postoperatively were not significantly different between the low-pain and high-pain cohorts in the PD and control groups but that may be an effect of small sample size. As expected, patients with lumbar disease who were scheduled to undergo lumbar procedures complained of higher pain than patients with anterior cervical disease. Future studies of patient satisfaction at their postoperative follow-up will help us better define the impact of preoperative pain in individuals with PD undergoing spine surgery.

Study limitations

Our study has several limitations. First, the retrospective nature of this study imposes limitations on patient selection, which explains the heterogeneous nature of our surgical population. Second, there was a gender disparity between the control and study groups. While the psychiatric disorder group consisted mostly of Caucasian females, our control group was dominated by Caucasian males. This is consistent with current data demonstrating that women are more likely than men to be diagnosed with a psychiatric disorder. The perception of pain may also be influenced by gender, and this may have affected the pain rating comparison between the PD and control groups.

## Conclusions

Spine surgery candidates with psychiatric disorders are more likely to have a higher preoperative pain rating. The incidence of narcotic medication intake is greater in patients with increased pain scores, although that does not appear to be the case in individuals with psychiatric disorders. It appears that psychiatric disorders may impact the degree of preoperative pain interference and the intake of narcotic medication independently from pain intensity ratings. These findings highlight the importance of detecting and treating depression and anxiety in patients suffering from spine disorders, regardless of surgical intervention planning.
